# Designing and Optimizing Electrode Materials for Energy Harvesting in CAPMIX Cells

**DOI:** 10.3390/nano14242031

**Published:** 2024-12-18

**Authors:** Belén Lobato, Samantha L. Flores, Lucía dos Santos-Gómez, Ana B. García, Alberto M. Pernía, Miguel J. Prieto, María G. Busto, Ana Arenillas

**Affiliations:** 1 Institute of Carbon Science and Technology (INCAR-CSIC), 33011 Oviedo, Spain; belen@incar.csic.es (B.L.); samantha.f@incar.csic.es (S.L.F.); anabgs@incar.csic.es (A.B.G.); 2 Department of Inorganic Chemistry, Crystallography and Mineralogy, University of Malaga, 29010 Málaga, Spain; 3 Department of Electrical Engineering, University of Oviedo, 33204 Gijón, Spain; amartinp@uniovi.es (A.M.P.); mike@uniovi.es (M.J.P.); gonzalezbusmaria@uniovi.es (M.G.B.)

**Keywords:** sol-gel synthesis, CAPMIX, carbon material, designed porosity, electrical conductivity

## Abstract

The growing demand for clean, decentralized energy has increased interest in blue energy, which generates power from water with different salt concentrations. Despite its potential as a renewable, low-cost energy source, optimizing electrode materials remains a challenge. This work presents a nanomaterial developed via microwave-assisted sol-gel methodology for blue energy applications, where ion diffusion and charge storage are critical. AX-7 carbon, designed for this study, features wide pores, enhancing ion diffusion. Compared to commercial NORIT carbon, AX-7 has a higher mesopore volume and external surface area, improving its overall performance. The synthesis process has been optimized and scaled up for evaluation in CAPMIX electrochemical cell stacks. Moreover, the lower series resistance (Rs) significantly boosts energy recovery, with AX-7 demonstrating superior performance. This advantage is especially evident during fresh-water cycles, where this material achieves significantly lower Rs compared to the commercial one.

## 1. Introduction

The search for sustainable and abundant energy alternatives is one of today’s priority objectives. To this end, it is essential to secure the energy sources that drive our current standard of living and development, avoiding damage to the environment and, if possible, preventing the use of critical materials that compromise the supply of such energy. In many cases, energy needs can be met with small or moderate supplies, making distributed generation highly attractive. This approach enhances supply security and reduces energy transmission losses. The abundant sea water, along with fresh-water streams that have been processed for human consumption and are then discharged into the sea at approximately 50% of their original volume, represent an untapped energy source. This process is known as blue energy, and it is usually referred to as capacitive mixing (CAPMIX) [1,2,3].

**Figure 1:**
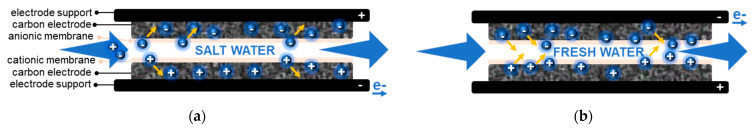
Scheme representing the phenomena in a CAPMIX cell during (**a**) the flow of salt water, followed by (**b**) the circulation of fresh water.

CAPMIX is a recent and innovative approach for electric power generation, utilizing a continuous flow of electrochemical process with water streams of different salinity [4,5,6,7]. This technology involves a set of techniques that vary the potential difference between the porous electrodes by interchanging the ionic content of the solution that comes into contact with them. This source has the potential to meet certain energy demands, offering about 2.5 MJ m^−3^ of water with minimal free-energy loss [8]. The operating principle of CAPMIX technology has been already proven at lab-scale [9] and it is shown in Figure 1. When salt water flows though the cell, the ion-exchange membranes promote the ions in the solution to diffuse to the electrodes (i.e., anions to the positively charged electrode and cations to the negatively charged one). This ions separation produces a voltage difference that induces electrons flow from one electrode to the other if a load is connected to them, producing a current and discharging the cell (Figure 1a). On the contrary, if fresh water (i.e., water from a river or treated water flow for disposal into the sea) flows through the cell, the ions previously adsorbed at the electrodes will diffuse again through the ion-exchange membrane into the flow, making the cell charged again, and electrons will circulate in the opposite direction if an external load is connected (Figure 1b).

Although this technology has already been validated, it still needs to be developed and optimized, specifically regarding electrode materials, to be effective in real operation. These active materials require the same physicochemical characteristics as those employed in desalination by capacitive deionization, due to the similar underlying principles [10,11,12]. Thus, they must have a combination of properties that are challenging to achieve in a single material, that is (i) large ion-accessible specific surface area, since the electrosorption capacity is related to this property; however, it should be noted that the whole surface area, as determined by classical methods, may not be fully accessible to ions; (ii) high ion mobility within the pore network; (iii) high electrochemical stability over the used pH and voltage, to prevent oxidation; and (iv) high electronic conductivity, in order to ensure a low-energy dissipation and low heating [13,14,15]. Particularly, the combination of high porosity (i.e., presence of feeder pores and high microporosity) and high electrical conductivity is especially difficult as they are counteracting properties. Thus, the basically non-porous materials with a very condensed and ordered tri-dimensional structure show excellent electrical conductivity. However, a high degree of porosity and an amorphous structure result in low electrical conductivity.

In addition to these requirements for proper operation, the necessary raw materials must be widely available (i.e., avoidance of critical materials) and the manufacturing process must be scalable and efficient. Most of the materials used for optimizing these electrodes are carbon-based [16,17,18,19]. Carbon materials are generally stable from a chemical standpoint and, furthermore, they are relatively easy to produce with very high specific surface areas. Among them, activated carbons are valued for their large surface area and porosity, which optimize ion adsorption capacity, and therefore, they have been one of the most studied materials in CAPMIX systems [20,21,22,23]. However, achieving an activated carbon material with optimal hierarchical porosity is very challenging, as obtaining a high volume of both mesoporosity and microporosity through an activation process is nearly impossible.

An alternative is to design and produce a synthetic carbon material with the desired nanoporosity along with other necessary properties. To that, sol-gel methodology allows one to design the physicochemical properties at nanoscale such as porosity, chemical composition, structure and morphology, among others, including the elimination of impurities that could disrupt the electrochemical process and affect electrode stability [24,25,26,27,28]. This process also allows enhanced electrical conductivity through the addition of specific additives to the precursor mixture, which are incorporated in the carbon matrix without compromising the desired porosity [29]. Traditionally, the sol-gel method involves a lengthy process and requires extreme operating conditions (i.e., temperature, pressure) to remove the solvent without modifying the nanoporous structure. However, a notable improvement has been demonstrated in previous studies by using microwave-heating throughout the whole sol-gel process (including polymerization, curing, and drying steps). This approach notably reduces the processing time by over 90% and allows for precise control of the designed hierarchical porosity [30,31]. Carbon materials prepared via microwave-assisted methods have been widely used, particularly as electrode materials in electrochemical energy devices such as supercapacitors, batteries, and fuel cells, due to their enhanced properties like high conductivity and tunable porosity [27,32]. However, these materials with customized properties have never been investigated for CAPMIX applications. This presents a significant opportunity to explore and develop new solutions that could optimize the performance of these devices.

In this work, a tailored synthetic carbon material, specifically designed to meet precise requirements, is employed as electrode for the CAPMIX cell. A microwave-assisted sol-gel methodology, followed by an activation process, is used for its synthesis, resulting in an activated carbon material with a highly controlled meso- and micropore structure, developed independently. The importance of the proper combination of the nanopores and carbon structure is revealed. This material also benefits from enhanced electrical conductivity due to the addition of graphene oxide to the precursor mixture. The advantages of the developed material in comparison to commercially available activated carbons that have been previously tested for the same application are also studied. Besides, this is the first work in which a tailored synthetic carbon is tested as electrode material in a prototype CAPMIX cell.

## 2. Materials and Methods

### 2.1. Preparation of the Synthetic Carbon Material

A mixture of resorcinol (R, 99% from Indspec) and formaldehyde (F, solution 37% stabilized with 10% of MeOH from Merck) were dissolved in water under stirring. The polymer was obtained using a molar ratio R/F of 0.5, and a dilution ratio (ratio of solvent and solid used) of 5.7 in order to obtain a carbon material with a porous size of about 10 nm [20]. Half of the necessary water for that dilution ratio was substituted by an aqueous suspension of graphene oxide, in order to introduce graphene into the polymeric structure of the material. A concentration of 5 mg mL^−1^ of commercial graphene oxide solution (Applynano, Alicante, Spain) was chosen because previous studies have demonstrated that this amount is sufficient to achieve the percolation threshold in graphene-doped carbon gels, leading to enhanced electrical conductivity, while remaining low enough to prevent aggregation and ensure uniform dispersion throughout the carbon matrix [33]. NaOH 1 M was used to adjust the pH to 6.5 and promote the polymerization reactions. The mixture was placed in a custom-designed multimode microwave oven (operating at a frequency of 2450 MHz, equipped with temperature control and magnetron power regulation) at 85 °C for 5 h to undergo the whole sol-gel process (i.e., polymerization, aging and drying steps) [28]. The resulting polymer was subjected to a one-step carbonization/activation process in a tubular furnace under CO_2_ atmosphere (100 mL min^−1^) at 1000 °C for 2 h to convert the organic material into carbon material through a controlled heating process in the absence of oxygen and obtain the desired material designed as AX-7. For comparative purposes, an activated carbon (Norit Super 30, NORIT, Amersfoort, The Netherlands) which is commercialized for electrodes of supercapacitors was also selected and tested in this application.

### 2.2. Physicochemical Characterization

The porous properties of the materials were evaluated by means of N_2_ adsorption–desorption isotherms at −196 °C (Tristar 3020 instrument, Micromeritics, Norcross, GA, USA). The samples were outgassed previously by heating at 120 °C under vacuum overnight. The BET equation was employed to calculate the specific surface area (S_BET_), whilst the t-plot method was used to evaluate the external surface area (S_EXT_). The total pore volume (V_T_) was calculated at the saturation point (i.e., p/p^0^ = 0.99), the micropore volume (V_MICRO_) was obtained from Dubinin–Radushkevich equation, and the difference with the total pore volume was attributed to the mesopore volume (V_MESO_). The pore size distribution was calculated by means of 2D-NLDFT-heterogeneous model (SAIEUS software V3.0) applied to the adsorption branch of the nitrogen adsorption isotherm. The micro/nano-metric surface morphology of the materials was examined by high-resolution transmission electron microscopy (HRTEM) using a JEOL (Croissy-sur-Seine, France) JEM-2100F microscope operated at an accelerating voltage of 200 kV, equipped with a field emission gun (FEG). The samples were dispersed in ethanol, sonicated, and sprayed on a carbon-coated copper grid and then allowed to air-dry. The chemical composition of both materials was also determined by elemental analysis performed with LECO-CHNS-932 and LECO-VTF-900 microanalyzers, and possible impurities were obtained by ICP-MS (Agilent, Madrid, Spain, ICP-MS 7700x). Prior ICP-MS evaluation, a digestion step, was conducted with HCl/HNO_3_/H_2_O in a ratio 3:2:1 for 50 min using a digestor (Milestone, Madrid, Spain, ETHOS-1). Electrical conductivity of the active materials was evaluated by the 4-point probe method (model SR-4-6L from Everbeing, Adler, Madrid, Spain).

### 2.3. Electrode Preparation, Electrochemical Measurements, and CAPMIX Cell Assembly

The commercial active-carbon NORIT as received and the synthetic carbon-gel AX-7 (pulverized at 30 Hz for 5 min in a mixer mill Retsch MM400 (Haan, Germany) and sieved to a particle size below 50 μm) were used as the active material for the electrode fabrication. The electrodes were prepared by mixing the active material selected, carbon black as the additive (CB, Super C65 from Imerys, Paris, France), and polyvinylidene fluoride (PVDF, from Aldrich, Darmstadt, Germany) dissolved in 1-methyl-2-pyrrolidone (NMP, reagent plus 99% from Sigma Aldrich, Darmstadt, Germany) as a binder to obtain a slurry. Through an extensive optimization process, various slurries with different component ratios were formulated and evaluated based on electrode stability, electrical conductivity, and tendency to delaminate. It was determined that the optimal slurry combination was 90 wt.% active material, 8 wt.% PVDF, and 2 wt.% CB in 1-methyl-2-pyrrolidone. The preparation process began with dissolving 2 wt.% of PVDF in pyrrolidone by mechanical stirring at 1000 rpm for 30 min in an Overhead Stirrer Eurostar 20 (IKA, Barcelona, Spain). Then, the correspondent mass of C65 was added to the PVDF solution while the stirring continued at 1000 rpm for 10 min. Finally, the mass of active material selected was incorporated and the stirring continued at 4000 rpm for 1 h. The obtained slurry was tape-casted onto the graphite support (isostatic graphite, quality 1949, 11 × 10 × 0.2 cm and manufacture by Mersen, Sant Feliu de Llobregat, Spain) used in the CAPMIX cell, covering a square centered surface of 8 × 8 cm. The tape-casting was performed using a standard doctor-blade and a monitored/automatic film applicator with a perforated vacuum table heated at 50 °C (Elcometer 4340, Elcometer, Barcelona, Spain). The deposited material was dried on the applicator at 100 °C for 1 h under vacuum and additionally overnight in a vacuum oven at 150 °C. Different electrode thicknesses were tested, 100, 150, 200, 300 and 400 μm, but based on the performance and charge-storage capacity, a final optimized thickness of 300 µm was selected for the electrodes to be studied in the cells. This thickness refers to the final thickness of the electrode after drying, and it was measured using a laser (Keyence LK-2101, Itasca, IL, USA) [34,35].

The electrical conductivity of the plain graphite support and the electrode/graphite-support assembly before electrochemical testing was also determined using the 4-point probe method in two different ways: (i) along the sample surface (i.e., the probes were placed on the horizontally positioned sample and separated 50 mm), and (ii) transversally through the support or electrode/support assembly (i.e., the probes were place on opposite sides of the workpiece to be measured, taking into account that the support has a thickness of 2 mm and the electrode 300 microns). “Rs assembly” denotes the static resistance of the electrode assembly before electrochemical activity, while “in operando” refers to the internal resistance of the cell during operation with a NaCl solution.

**Figure 2:**
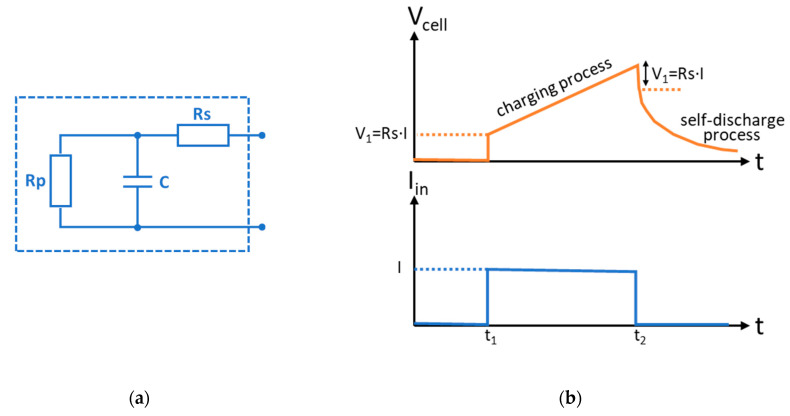
(**a**) Lumped-parameter model and (**b**) experimental test for CAPMIX cell characterization.

In addition to the previously mentioned graphitic-support and the electrode, anionic and cationic membranes (Fumasep FAS-30 and FKS-30, respectively, both 10 cm × 10 cm from Fuel Cell Store, TX, USA) as well as a silicone/polypropylene spacer-gasket (ED 64-102-086, 0.45 mm thick, from PC Cell, Marpingen, Germany) were used in the assembly of the CAPMIX cell at room temperature and pressure based on Donnan Potential [36,37]. This cell shows electrical behavior similar to that of a supercapacitor, as ions are adsorbed onto the electrodes, forming an electrical double layer. Therefore, a classical lumped-parameter model [38,39] can be defined (Figure 2a), which includes a capacitor (C) where charge is stored, a series resistor (Rs), that represents ohmic losses when extracting the energy out of the capacitor, and a parallel resistor (Rp) that justifies the self-discharge of the cell. In order to determine the characteristic parameters of the CAPMIX cell with the different active materials in the electrode (i.e., C, Rs, and Rp), saline water of different concentrations (0.1, 0.3, and 0.6 mol L^−1^, prepared by adding sodium chloride to fresh water) was pumped into the cell. At a constant current (I), the voltage will increase (Figure 2b), due to the diffusion of the ions to the electrodes, more or less in a linear way; this will allow us to calculate the equivalent capacitance (C). When the current (I) is interrupted, a self-discharge process can be observed defining the value of Rp. Finally, Rs can be calculated from the voltage variation occurring when the current starts or stops (Rs = V_1_/I in Figure 2b). On the other hand, Rp can be calculated from the self-discharge process (Figure 2b) considering the following equation Rp = Δt/(C·Ln(V_C1_/V_C2_)). In this work, the Rp values obtained for NORIT commercial carbon in salt and fresh water were 28 and 16 ohms, respectively. Whilst for the AX-7 sample, it was 40 ohms in both salt and fresh water.

## 3. Results

The active materials selected for the electrode fabrication were previously physicochemically characterized and then used to fabricate the electrodes to be tested in the CAPMIX cell.

### 3.1. Electrode Material Properties

The elemental analysis shown in Table 1 reveals the similarity in terms of chemical composition of the active electrode materials studied: the synthetic carbon (AX-7) designed specifically for this work and the reference commercial activated carbon (NORIT). Both materials are composed mainly by carbon and there are no content impurities that may influence their electrical conductivity or the electrode-based stability during cell operation. It is worth mentioning that, unlike commercial carbon, which undergoes acid washing to remove impurities, the AX-7 obtained by the sol-gel method is free of impurities because of the highly controlled process and, therefore, does not require any post-synthesis treatment for its suitability, which is a great advantage in terms of production operating costs.

**Figure 3:**
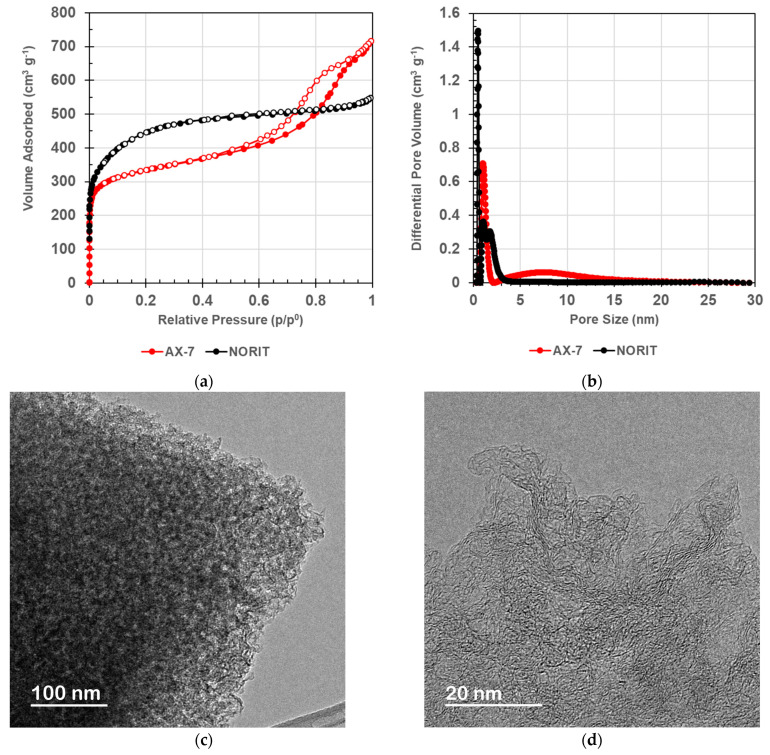
(**a**) N_2_ adsorption–desorption isotherms of the active materials used in the electrodes; (**b**) Pore size distribution obtained from 2D-NLDFT Heterogeneous Surface model; (**c**,**d**) HRTEM of AX-7 synthetic material evaluated in this work.

The main differences between NORIT and AX-7 carbons are their porous structure. As can be seen in Figure 3a, the N_2_ adsorption–desorption isotherms present completely different shapes, indicating different porosity. NORIT exhibits a type I isotherm according to IUPAC classification, typical for microporous materials. The high increase in nitrogen adsorption at low relative pressures indicates a high volume of micropores, and the adsorbed volume remains nearly constant at moderate and high relative pressure, indicating the absence of meso- and macroporosity. Only the wide knee at low relative pressure points denotes the presence of very narrow mesopores. The AX-7 shows a type II isotherm, with a combination of (i) an increase in adsorbed volume at low relative pressures revealing the presence of micropores and (ii) an increase in adsorbed volume at moderate relative pressures and a hysteresis loop, clearly showing the existence of mesoporosity. These different features regarding the nanoporosity are corroborated by the results of the pore size distribution in Figure 3b. Thus, NORIT carbon presents only narrow pores with widths no greater than 3 nm, whereas AX-7 carbon shows narrow pores for charge storage and wider pores (i.e., feeder pores) with a maximum width of 7 nm, which improve the diffusion of ions, making the microporosity more accessible.

Table 2 shows the porous parameters of the two electrode-active materials. NORIT carbon has a higher micropore volume and, therefore, a larger specific surface area than AX-7 material. However, the designed carbon presents a much higher volume of mesopores (0.78 cm^3^ g^−1^) and, in consequence, the total pore volume is also higher than that of the commercial NORIT active carbon. Moreover, the external surface area, S_EXT_, of AX-7 is much higher than that of NORIT (424 vs. 78 m^2^ g^−1^, Table 2). This difference is very relevant because the optimum electrode material for CAPMIX cells should present high active surface area for ions adsorption, and the S_EXT_, which is the most available surface area in the nanoporous structure of the material, is closer to this concept than other classical models for specific surface area calculation such as that calculated mainly based on microporosity (i.e., S_BET_).

The electrical conductivity of the active materials is shown in Table 3, with values of 120 and 45 S m^−1^ for AX-7 and NORIT, respectively. Unlike conventional carbon materials, in which porosity often hinders electrical conductivity, the incorporation of a graphene oxide suspension into the polymer precursor mixture of AX-7 effectively addresses this issue. The graphene oxide sheets were perfectly integrated into the polymeric structure (Figure 3c,d), optimizing the material’s properties for its intended application. Throughout the post-synthesis heat treatment, the oxygen functionalities were mostly eliminated and the final material (AX-7) exhibits a C-structure composed by reduced-graphene integrated in the amorphous carbon nanostructure [29,33]. As a result, and despite the high porosity, the electrical conductivity of this material through the walls of the nanoporous structure is much higher than that usually showed by the activated carbons. Specifically, the electrical conductivity of AX-7 is nearly three times larger than that measured for NORIT (Table 3).

**Figure 4:**
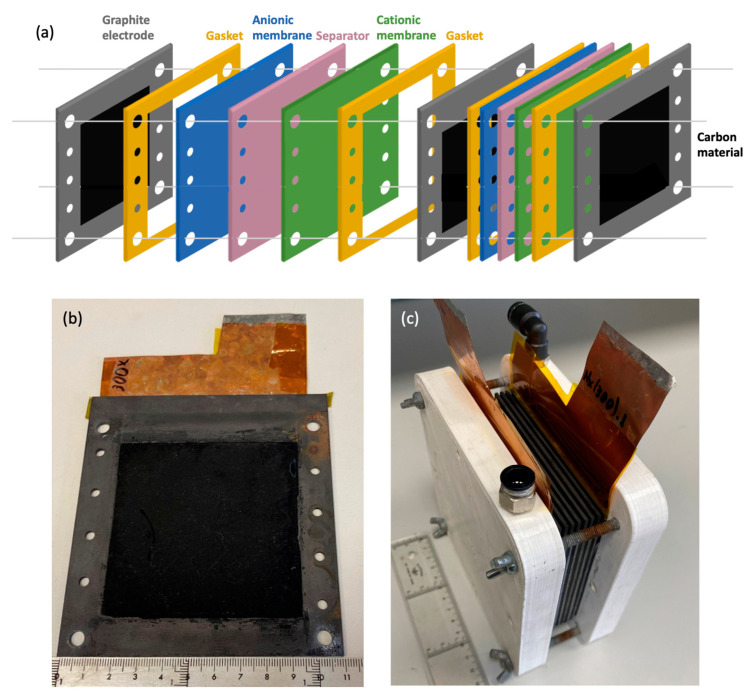
(**a**) Schematic representation of the CAPMIX cell, illustrating the arrangement of components. (**b**) Carbon-based electrodes for the CAPMIX cell and (**c**) cell stack assembly.

The nature and proportion of active material, binder and additive, are crucial for electrode performance, influencing the desired good electrical conductivity of the electrode assembly. Moreover, the contact of the electrode and the current collector (i.e., graphite support) will be also relevant (see Figure 4b). It has to be mentioned that the measurements were always performed at room temperature (ca. 20 °C) and atmospheric pressure. Due to this, the electrical conductivity of the electrode assembly was measured using the 4-point probe method both horizontally (H) over the surface of the electrode and transversally (T) through the assembly (Table 3). The surface and the transversal electrical conductivities of the electrode assembly using AX-7 active material are more than two times and nearly four times higher, respectively, compared to those with the commercial active-carbon NORIT. This means that not only does the designed active material AX-7 present better properties (i.e., more adequate porosity and higher electrical conductivity) but also both the electrode composition and the electrode/current collector contact are well optimized, resulting in an electrode assembly ready for operational testing (Figure 4).

### 3.2. CAPMIX Cell Performance

**Figure 5:**
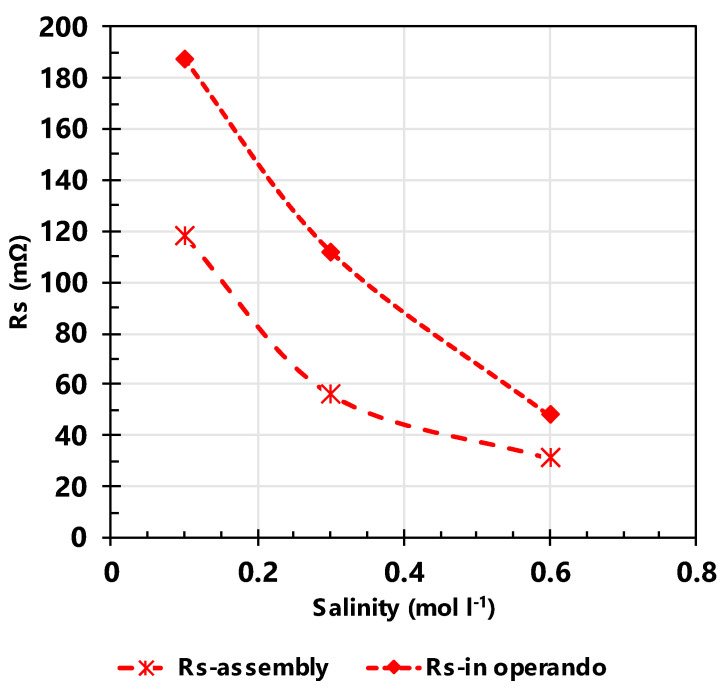
Series resistance (Rs) of the cell as a function of salinity of the water without and within operation (constant flow of 240 cm^3^ min^−1^), using electrodes of 300 μm with AX-7 as active material.

Two single CAPMIX cells were tested following the voltage–time variation protocol described in Figure 2b using the AX-7 carbon, and the commercial NORIT one, for comparative purposes. Different salinities were examined in the single CAPMIX cell using AX-7 as electrode-active material. Figure 5 shows that the Rs of the system is higher in the case of the cell during operation. The continuous flow of 240 cm^3^ min^−1^ of salty water obviously influences the efficiency of the system. However, in both cases (i.e., continuous operating or static measurement), the series resistance decreases with the increase in the salinity of the water. The higher concentration of ions favors the electrical conductivity of the system and the cell behavior, leading to a decrease in the series resistance. Therefore, a concentration of 0.6 M of salt in the water is used for all subsequent analyses conducted with the CAPMIX cell.

**Figure 6:**
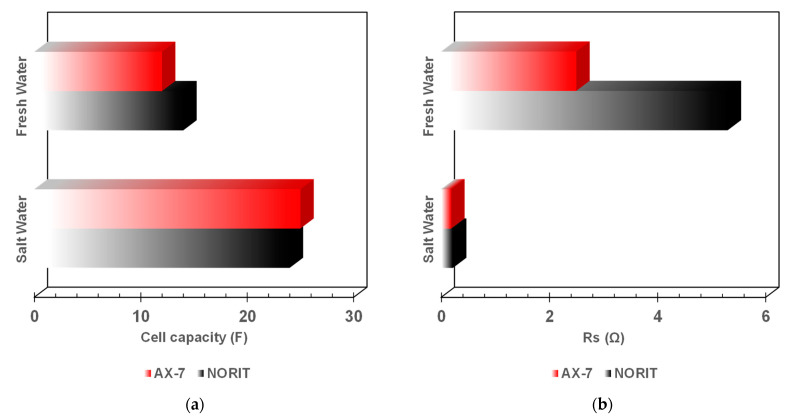
Comparison of the cell capacity (**a**) and series resistance (**b**) between both active materials evaluated and operating with both fresh and salt water (0.6 M). In all cases, the electrode thickness was 300 μm, the current was 100 mA during charge and 10 mA during discharge, and the water flow was 240 cm^3^ min^−1^.

Finally, the single cells were tested under operating conditions with a constant flow of 240 cm^3^ min^−1^ of salt water 0.6 M and a charge current of 100 mA; the discharge current was 10 mA after circulating fresh water. The electrodes, made with either NORIT or AX-7 as active materials, were in all cases of 300 µm thickness. Thicker electrodes increase charge capacity but can reduce cell efficiency due to poor salt and water diffusion, higher electrical resistance, and potential delamination issues. The main cell parameters obtained from these operation tests are presented in Figure 6.

Figure 6a shows the cell capacity during the salt-water (0.6 M) and fresh-water (i.e., water with 300 μS) cycles. Interestingly, the capacity under fresh water is larger in the case of using the commercial carbon NORIT; this is probably due to the higher specific surface area of this carbon (i.e., 1615 m^2^ g^−1^ vs. 1248 m^2^ g^−1^ of AX-7, Table 2), as it is commonly accepted in double-layer charge-storage phenomena (the higher the surface area, the larger the charge-storage capacity). However, when salt water is used, the presence of feeder pores plays a relevant role in the diffusion and interaction of solvated ions, and the synthetic carbon AX-7 exhibits better performance due to the presence of a high volume of these feeder pores. Moreover, although the capacitance is a relevant parameter in this kind of device, the series resistance is of equal or greater importance as this is a key factor for the energy-generated recovery. In this case, the Rs is in the order of mΩ, although higher in the case of commercial carbon, for the salt-water cycle. However, during the fresh-water cycle, the measured Rs is in the order of Ω, being double for the carbon NORIT (Figure 6b).

Based on the tests performed with single cells, a stack was built with 10 cells in parallel (see Figure 4c), using both the AX-7 and the NORIT, in order to have 1 V maximum. This CAPMIX stack features the same electrodes shown in Figure 4b at both ends, where the carbon material is deposited on only one side, while the intermediate graphite supports have electrodes deposited on both sides. The microwave-assisted sol-gel process is able to produce high amounts of the desired material so such a prototype was able to be tested. Stacking did not penalize the performance and remains proportionate to the number of cells [40].

## 4. Discussion

The sol-gel process allows researchers to design and produce a carbon synthetic material that combines all the properties required for a given application. These particular carbon material properties are not achievable by other non-synthetic routes such as the widely used biomass waste carbonization and activation. In this context, and taking advantage of the microwave-assisted sol-gel process previously developed, it is possible to design and mass produce a carbon gel with the required specifications to be used as electrode-active material for CAPMIX cells. The resulting material presents a combination of (i) mesopores, also called feeder pores, to promote the diffusion of ions and make the surface area more available; (ii) high microporosity and surface area to improve charge-storage capacity; and (iii) high electrical conductivity (i.e., 120 S m^−1^) and no impurities, being composed mainly by C (>95 wt.%). Similar materials have already exhibited very good performance as electrodes for supercapacitors [41]. The similarity between the basic fundamentals of both applications (i.e., energy storage in supercapacitors and energy harvesting by CAPMIX cells) provides the necessary tools to design a material with the suitable properties for a good performance in energy supply be means of CAPMIX cells.

In this specific application, it is not only necessary to maximize surface area to enhance charge-storage capacity, but also to optimize total porosity. An appropriate combination of feeder pores (i.e., mesoporosity) and microporosity is necessary. The former will favor the diffusion of ions (solvated or partially solvated) in addition to improving the availability of the microporosity. The second is directly related to the amount of charge-storage capacity and should obviously be maximized. It is worth mentioning that this combination of micro-mesoporosity is very difficult to obtain by classical activation processes. In addition, the feasibility and yield of the production processes of these nanomaterials must always be taken into account; steps such as activation, acid washing, etc., have an important impact on both efficiency and cost. The methodology presented in this work displays numerous advantages, since the mesoporosity can be designed during the polymerization process, independently of the microporosity that develops in a post-synthesis process. The chemical composition is fully controlled and unwanted impurities are avoided without any additional washing step. Moreover, maximizing porosity and electrical conductivity is a difficult task, as they are antagonistic properties. It is only possible by designing a synthetic nanomaterial with the desired porosity but with pore walls formed by a carbon nanostructure that favors electrical conductivity.

Electrical conductivity is necessary to minimize the associated system resistances (Rs) that would penalize the recovery of harvested energy. The Rs is influenced by several aspects. Some of them are related to the cell spare parts (i.e., connectors, ionic membranes, etc.), some to the operating conditions of the cell (i.e., current, etc.), but all of these are out of the scope of this work. Only some parameters related to the active nanomaterials used in the electrode were evaluated, and this is widely considered as a key point for the development of this blue energy. Several electrode thicknesses ranging from 100 to 400 μm were tested. The greater the thickness, the higher the active material content and thus the higher the charge capacity. However, electrodes that are too thick present more difficulties for salt- and fresh-water diffusion, in addition to a higher electrical resistance, which would result in a loss of efficiency of the cell. The optimum thickness depends on the properties of the active material used, including particle size, porosity, etc. which influence the packing of the electrode. Therefore, an optimization of this parameter was carried out with the commercial carbon NORIT and then used with the synthetic material AX-7 for comparative purposes. From these experiments, it was inferred that an electrode thickness of 300 μm was the optimum for this application. Similarly, an optimization of the electrode composition was performed regarding the amounts of binder and CB additive since they depend on the physicochemical characteristics of the active material and would affect the electrode stability and performance. Again, this optimization was made with the commercial carbon, and then used in the AX-7 nanomaterial-based electrodes fabrication. This implies that the synthetic nanomaterial designed in this work still has much room for improvement, not only optimizing the mesopore size, or maximizing the micropore volume by further activation, or increasing the electrical conductivity by increasing the graphene oxide content in the precursor solution, but also optimizing the composition and thickness of the electrodes made based on it. Furthermore, there are some studies that mention that the surface chemistry of the active material may be modified in order to improve the performance of the electrodes in this application [42]. This is an aspect that was out of the scope of this work but that should be taken into account, as the synthetic materials can be physicochemically designed.

The optimal electrode composition and thickness were then used for comparison between the commercial carbon and the synthetic AX-7. The results reveal that the mesopores favor the capacity values in salt-water flow, but, more important, the Rs is minimized using AX-7 in the electrode, besides lower charge/discharge times, due to the high electrical conductivity. In sum, the synthetic material designed by sol-gel methodology exhibits better performance in CAPMIX cells and therefore higher energy recovery. Based on the tests performed with single cells, a stack was built with 10 cells in parallel. The stack’s performance remained consistent with the number of cells, showing no performance penalty. Based on theoretical projections, it is estimated that the system could achieve an energy output of approximately 250 kJ day^−1^, with peak currents near 10 A, using the AX-7 nanomaterial and a cell volume of 3 L. This would represent a twofold improvement over the figures provided by commercial NORIT carbon material.

## 5. Conclusions

In conclusion, the increasing demand for clean, decentralized energy has underscored the potential of blue energy, which generates power from water with varying salt concentrations. Despite its potential as a renewable and low-cost energy source, optimizing electrode materials remains crucial. This study highlights the effectiveness of the microwave-assisted sol-gel methodology as a competitive and efficient approach for designing and mass-producing synthetic carbon materials with optimized physicochemical properties for power generation from water streams of different salinity. The AX-7 carbon nanomaterial, developed in this work, features a strategic combination of mesoporosity (0.78 cm^3^ g^−1^) and external surface area (424 m^2^ g^−1^), which enhances ion diffusion compared to commercial NORIT carbon. Notably, AX-7′s lower series resistance (Rs) significantly improves energy recovery, particularly in fresh-water cycles. The optimized synthesis process has been scaled up and tested in CAPMIX electrochemical cell stacks, showing exceptional performance and confirming that the synthetic carbon-gel AX-7, designed specifically to meet the cell requirements, is suitable for blue energy applications.

## Figures and Tables

**Table 1:** Chemical analysis of the carbon materials used as active material for the electrodes.

Material	C wt.%	H wt.%	O wt.%	N wt.%	S wt.%
AX-7 *	95.4	0.6	3.1	0.9	0.0
NORIT	95.7	0.5	2.8	0.9	0.1

* ICP-MS of Na, Mg, Al, K, Ca, V, Cr, Mn, Fe, Co, Ni, Cu, Zn < 0.5 wt.%.

**Table 2:** Porous parameters of the electrode-active materials.

Materials	V_T_ (cm^3^ g^−1^)	V_MICRO_ (cm^3^ g^−1^)	V_MESO_ (cm^3^ g^−1^)	S_BET_ (m^2^ g^−1^)	S_EXT_ (m^2^ g^−1^)
AX-7	1.11	0.33	0.78	1248	424
NORIT	0.85	0.71	0.14	1615	78

**Table 3:** Electrical conductivity of the electrode-active materials (AX-7, NORIT), graphite support, and electrode assembly, measured in horizontal and transversal mode.

Material	Electrical Conductivity (S m^−1^, ±5)	Electrode Assembly	
		H-Measurement (mS)	T-Measurement (mS)
AX-7	120	83	222
NORIT	45	34	62
Graphite support	75,758	83,333	10,000

## Data Availability

The original contributions presented in the study are included in the article, further inquiries can be directed to the corresponding author/s.
